# Draft Genome Sequence of the Necrotrophic Plant-Pathogenic Bacterium Pectobacterium carotovorum subsp. carotovorum Strain LMG 2410

**DOI:** 10.1128/MRA.00614-19

**Published:** 2019-08-01

**Authors:** William M. Rooney, Marta Wojnowska, Daniel Walker

**Affiliations:** a Institute of Infection, Immunity and Inflammation, College of Medical, Veterinary and Life Sciences, University of Glasgow, Glasgow, United Kingdom; b Plant Science Group, Institute of Molecular, Cell and Systems Biology, University of Glasgow, Glasgow, United Kingdom; Portland State University

## Abstract

Here, we report the draft genome sequence of Pectobacterium carotovorum subsp. carotovorum strain LMG 2410, isolated from cucumber in the United Kingdom. The draft genome is 4,773,000 bp, with a G+C content of 51.9%, and carries a total of 4,536 coding sequences.

## ANNOUNCEMENT

Pectobacterium carotovorum subsp. carotovorum is a Gram-negative bacterial plant pathogen, possesses a broad host range, including many important crop species, and is a causative agent of soft rot (1). Soft rot disease symptoms arise from an arsenal of secreted cell wall-degrading enzymes, such as pectinases, cellulases, and xylanases, which allow the bacterium to necrotize its host (2, 3). One of the *P. carotovorum* subsp. *carotovorum* strains isolated from cucumber in the United Kingdom has been used to investigate the import of the abundant plant protein ferredoxin from which *P. carotovorum* can acquire iron for growth (4–6). Iron acquisition from ferredoxin is mediated by the ferredoxin uptake system (Fus), of which the outer membrane TonB-dependent receptor, FusA, and the periplasmic M16 protease FusC have been demonstrated to be essential for this process (4, 5). This study used this strain to generate a draft genome which can be a tool to further dissect this protein import pathway.

The genomic DNA of *P. carotovorum* subsp. *carotovorum* was extracted from a freshly grown single colony grown in lysogeny broth (5 g liter^−1^ peptone, 5 g liter^−1^ tryptone, 10 g liter^−1^ NaCl [pH 7.5]) using the GenElute bacterial genomic DNA kit (Sigma-Aldrich, Dorset, UK). Library preparation was performed using the TruSeq DNA Nano kit and size selected for the large fragment size (Illumina, CA, USA). Sequencing was performed using the Illumina MiSeq 500 platform with a 2 × 300-bp paired-end protocol with 50× read depth. A total of 504,901 raw reads were trimmed (with a quality score limit of 0.05) and assembled *de novo* using CLC Genomics Workbench version 7.5.2 (CLC bio, Denmark) using the default settings, which generated 132 contigs with a total length of 4,783,145 bp (G+C content, 51.9%), an *N*_50_ value of 339,669 bp, and minimum and maximum contig lengths of 140 and 879,473 bp, respectively. Genome annotation of the assembled contigs was performed using the Rapid Annotations using Subsystems Technology (RAST) server, which identified 4,536 coding sequences, 70 tRNAs, and 28 rRNAs (7–9). From the RAST subsystem annotations, 41 genes were identified as being involved in iron acquisition and metabolism. Furthermore, from the keyword searches of the annotated genome, we identified 6 genes encoding TonB-like proteins, 5 sets of *exbBD* genes, and two genes encoding M16 proteases, including FusC (5).

In conclusion, we have reported the draft genome sequence of *P. carotovorum* subsp. *carotovorum,* a bacterium originally isolated from cucumber in the United Kingdom.

### Data availability.

This draft genome project has been deposited at DDBJ/EMBL/GenBank under the accession number VBUA00000000 (BioProject number PRJNA543207 and BioSample number SAMN11658211). The version described in this paper is the first version, VBUA01000000.
